# Rebellion, curiosity, and perseverance: a conversation with Maria Angela Franceschini

**DOI:** 10.1117/1.NPh.10.1.010401

**Published:** 2023-01-09

**Authors:** Erin M. Buckley

**Affiliations:** a Emory University, Georgia Institute of Technology, Wallace H. Coulter Department of Biomedical Engineering, Atlanta, Georgia, United States; b Emory University, Department of Pediatrics, Atlanta, Georgia, United States

## Abstract

Neurophotonics Associate Editor Erin M. Buckley (Georgia Institute of Technology and Emory University) interviewed her colleague Maria Angela Franceschini, professor at the Athinoula A. Martinos Center for Biomedical Imaging, Massachusetts General Hospital, Harvard Medical School, and recognized leader in the field of diffuse optical imaging in both neuroscience and clinical neuro-monitoring applications.

**Figure:**
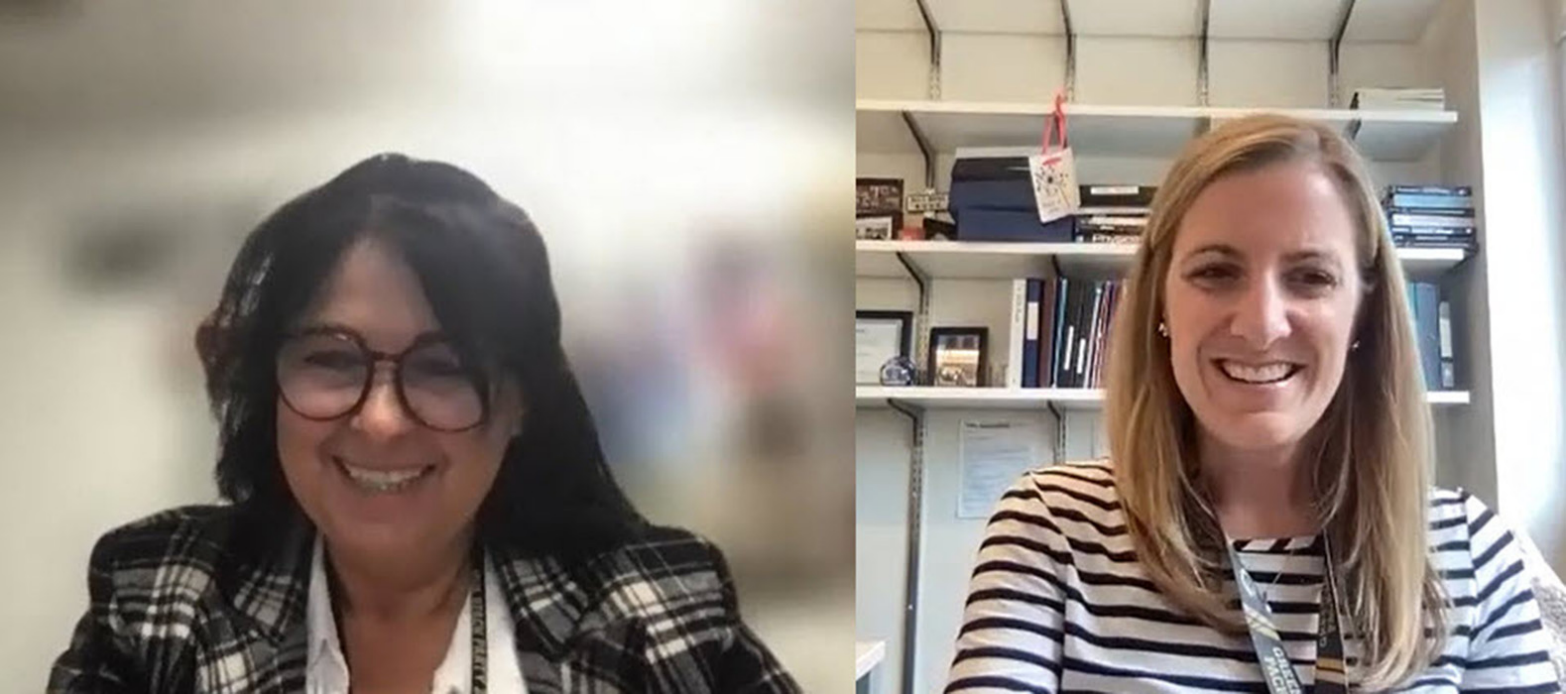
Neurophotonics *associate editor Dr. Erin M. Buckley (right) interviewed Dr. Maria Angela Franceschini, professor at the Athinoula A. Martinos Center for Biomedical Imaging, Massachusetts General Hospital, Harvard Medical School, and* *recognized leader in the field of diffuse optical imaging in both neuroscience and clinical neuro-monitoring applications. Readers are also invited to view the interview in video format*, https://doi.org/10.1117/1.NPh.10.1.010401.s1.

Erin M. Buckley: Hello, everyone. My name is Erin Buckley and I’m an assistant professor in the Department of Biomedical Engineering at Georgia Tech and Emory. Today I am honored to introduce Dr. Maria Angela Franceschini here as our guest at *Neurophotonics*. Dr. Franceschini is a professor in radiology at Harvard Medical School who is a world-renowned pioneer and leader in the field of diffuse optical imaging and spectroscopy for brain applications. She currently serves as the President of the Society for Functional Near-infrared Spectroscopy. She’s a Fellow for Optica and AIMBE. She’s a distinguished investigator in the Academy of Radiology Research. She’s the author of over 100 peer-reviewed journal articles, numerous patents, and several commercial ventures. So welcome, Mari!

Maria Angela Franceschini: Thank you, Erin. Thank you for the introduction. Nice to see you!

Buckley: You too! So, I was hoping we could start off by just having you tell us a little bit about your career trajectory. I know grew up in a small town in Italy and you did your PhD there in physics. But also – knowing you, you’re very creative and artsy – I’m wondering what drew you to the sciences and physics, in particular, initially to get your PhD?

Franceschini: Oh my, it’s a long story. I grew up in this small town in Tuscany, Cortona. It is a beautiful, touristic town, but it kind of gets boring. So, for me, it was important to move to Florence and get to the university. Also, I come from a family where everybody’s MDs, doctors, or pharmacists. And so I was a rebel – my rebellion was: I want to do physics.

Buckley: So rebellious, Mari!

Franceschini: I have a tattoo that is written “Science.”

Buckley: Oh, that’s awesome!

Franceschini: I’m not a real rebel. But I guess I had to be different. I studied physics. Florence is a beautiful place. Where the university back then was in the Galileo Villa where Galileo worked. Really nice. But physics was not me. It was so boring! and I was like: why did I choose Physics? I was doing fine, I was surviving in the university, so I continued. And definitely, I couldn’t do particle physics, nuclear physics, so I steered toward lasers. Laser was a little bit more interesting, and application of laser in medicine, so my thesis was on how to destroy urinary stones with excimer lasers.

Buckley: Oh, interesting!

Franceschini: I built this big laser system. That is how I wanted to stay away from medicine, and then I ended up getting back close to medicine.

Buckley: Came back into it, right?

Franceschini: There was no way I could miss it.

Buckley: So you joined the diffuse optics field in the early ’90s, right? In your postdoc?

Franceschini: Yeah, it was just very random. So, what happened was I’d just graduated and there were not many prospects for me in Italy back then. I was trying to see if there was any opening at the Centro Nazionale delle Recerche there in Florence, and I was working without being paid. There was a more senior researcher working with me that had married an American. She tried to live in Florence with him, but she didn’t like it. She went back to the US. And so he applied for jobs in the US. Enrico Gratton wanted to hire him and he planned to come visit us in Italy during one of his visits to Italy for other reasons, and he wanted to hire him. But by then they had divorced so he didn’t want to go to the US. And when Enrico came, he said why don’t you hire her? She just graduated, she’s good, she can do things. And I took the chance and said, why not? Let’s try and see how it is. I knew nothing about diffuse optics. So before going, I had like a month and a half, and I studied all the publications. There were not that many back then, but all Britton Chance, Mike Patterson, and so on, David Delpy. And I came to the States in January ’93, 30 years ago almost.

Buckley: Wow, so this was in Illinois? Was it University of Illinois?

Franceschini: Urbana–Champaign in Illinois. Big difference with Florence!

Buckley: Yes, quite a difference. So, your postdoc – you did a lot of the really pivotal early work on diffuse optical spectroscopy and separating out absorption from scattering. And then, after your postdoc, you transitioned to faculty at Tufts, correct? Briefly?

Franceschini: It was my ex-husband that got the faculty position. I just followed. So, they gave me a research, nontenure position. But it allowed me to apply for grants and I got an R01. And then, because of my interest in medical applications, I moved to MGH. And at Martinos, if you have a grant and you can self-support yourself, they’re very open to having you, if there is a common interest. So, I started moving there – I started working part time there in 2000. And then, I grew there because there were many more chances for me to apply NIRS and DCS for clinical applications.

Buckley: I guess probably right around that time is when you started really honing in on your focus of studying the brain. And so, what drew you to the brain?

Franceschini: We started with muscle and breast cancer. I found it more boring, less interesting, I guess. Brain was a big unknown back then. It was right when fMRI had started. It was when there were all these new theories for why there is big increase in blood flow in BOLD signals. There were a lot of open questions. It was very entailing. I remember in 2001, 2002, I had done – 2001, probably – I had a movie of functional activation and they invited me at the Human Brain Mapping. At the same time, there was Logothetis showing his movies of fMRI applications. So I was drawn by this blooming, growing field right then.

Buckley: Yeah! Well, and at the time, was the Martinos Center what it is today? It’s such a large MRI –

Franceschini: It was MRI. It was already Bruce Rosen. It was much much smaller, so we knew everybody. They were just starting building their MEG system. Yeah, it was way smaller. It was the period where we had parties, and still we could have parties at Bruce Rosen’s house. Now it would be impossible.

Buckley: But I guess, were there other people there who were also doing brain research who were stimulating your research interests?

Franceschini: Oh, of course. I was working with David Boas, my husband, he was already working there. He started the group. It was a great period. There was Joe Culver, Andy Dunn. Elizabeth Hillman came a little bit later. Anna Devor was there. So we were all together working with optical methods, either microscopy or diffuse optics, in the same lab. It was a really good time.

Buckley: Yeah, it sounds amazing. And now you’ve all gone on to be huge names in the field. What a unique opportunity!

Franceschini: That’s true.

Buckley: You’ve done many things, but I would say that you have a very large body of highly cited work on infants and the developing brain. And I’m curious if your research interests and children overlapped with when you had children of your own? Did they fuel each other?

Franceschini: No, Lisa was born much earlier. She was born in ’95.

Buckley: Your daughter, Lisa.

Franceschini: Yeah. No, it was more about – I thought it’s much easier to measure babies, especially infants. I was competing with fMRI back then, and MRI can do a lot for functional adults but can do very little for tiny babies. So I saw that as one of the most promising applications for NIRS. And of course, there was also interest, of course I thought it was important, but also there was a need – a higher need there – to have a neuro-monitoring system for babies, because there was no other good technologies that could do much.

Now, the technology is much better for NIRS, so we can also go back to adults and do things that MRI cannot do. But back then, the systems were very few channels and very simple.

Buckley: Right. Well, I imagine, too, the clinical environment at Brigham Women’s and –

Franceschini: Children’s…

Buckley: And Children’s – lots of babies around there!

Franceschini: I actually started at MGH. And again, another important issue is if you have the right collaborators. Ellen Grant was definitely a big collaborator for me, that really was pushing the technology for babies and understanding the needs. She was at MGH until 2009 – something like that – and then she moved to Children’s Hospital and I followed her there, because you need a strong clinical collaborator to be able to approach this population. And then, after a while, I also expanded to Brigham and now we’re also back at MGH.

Buckley: It’s interesting as you’re naming a bunch of these people, Ellen, and then, when you named the people who were with you back in the early days of MGH, you named Anna Devor and Elizabeth Hillman, you’re naming a lot of females. But I would say, in general, you grew up in science at a time when the field was – still is, in many ways – largely male dominated. So I’m wondering if you could share what challenges that brought on for you and also what opportunities potentially…

Franceschini: So, there was this period that I did – when I hired you - I wanted to hire only female postdocs! There was that bit, definitely. Now, lately, it’s kind of impossible, so I ended up with three male postdocs. And then, Stefan (Carp) hired a female and I was so envious! So, they still exist, it was not much chance at the moment I had the opening. But yeah, definitely the female point of view in research, the female way they dedicate to the work – I really like and it’s easier interaction. Maybe because I’m a female, it’s easier to interact with a female. But I cannot say anything’s wrong with my three guys over there. They are all exceptional.

Buckley: I mean, I guess I would say, thank you for paving the way and setting an example for younger researchers like myself about what it means to be a successful woman in the field. That must have been challenging. I imagine you didn’t have as many female role models when you were coming up.

Franceschini: No. Maybe my high school math teacher, but it was limited. Definitely not in physics in Florence. Postdoc with Enrico. Enrico is amazing. Enrico Gratton. But yeah, he’s a male figure, Bruce Rosen is a male figure. Yeah, no I didn’t. But I didn’t mind. I didn’t see it as a difference. It doesn’t really matter. The important is that is a person that values you. Not because you’re male or female, but for your way of thinking and doing things. I was lucky in that. I always found people that were supportive.

Buckley: Right. I feel like I’ve had a very similar experience and have found very supportive people. But it does really help to have positive role models to show you the way, who look like you, right? I appreciate it. So, let’s see, switching gears a little bit. In 2020, right before COVID shut the world down, you were diagnosed with lung cancer. And you’ve spoken very openly about the experience on social media. And I was just wondering if you could share some of the ordeal with the listeners. I know it actually started with a persistent cough that you noticed at (SPIE) Photonics West.

Franceschini: Yeah, it was February 2020 and the last few days of the meeting I started coughing. And I got this cold. Came back to Boston and the cold was not going away. And I was supposed to go ski and I said, OK, let me go to the doctor. Maybe I have COVID. It was the period, middle of February, when COVID was starting and we didn’t know exactly what it was and what it wasn’t. So I went to MGH walk-in clinic. They said let’s do a chest X-ray. And there it was, this ‘golf ball’ in my lung! It was pretty clear what it was. And yeah, it’s difficult. It’s an awful thing. It changes you totally.

At the beginning, I thought, OK, I’m dead. This is a death sentence. Then surgery, chemotherapy, radiation – it all feels surreal now. You don’t have choices. You just do things. And this was all during the peak of COVID in 2020, so it was a very very surreal experience. And definitely, I got traumatized by all that. And the thing now I realize, I have to live with it. It is not a disease that goes away. So even if I’ve no evidence of disease in my scans every three or four months, nobody will say “you’re cancer-free.” Right now, I’m with this targeted therapy that is supposed to last for three years. So I have one more year left on this targeted therapy. And they just published in September the results, and they’ve reached the cancer-free survival period that is 66 months. So, when you approach the five year since your diagnosis, instead of saying, OK, I’m done, then is when they will have to make more frequent scans and surveillance, because it’s when the cancer would be back. Mentally, it is so crippling the idea that you have a future, but you don’t have a future. You don’t know what’s going – every few months, every whatever, it’s not going away. You feel normal. You are fine. You are functioning. But there is this always with you. And it’s not that easy.

Buckley: Oh, that’s very rough. At this stage, I guess all of that happened pretty quickly. So you’ve been – I mean, you’re in the last year of your treatment. So, you’ve been back at work and fully functioning for over a year now, correct?

Franceschini: I mean, the only period I couldn’t work was during chemotherapy. That’s awful. You can’t really work with chemotherapy. The rest, yeah – in August 2020, I was back in the lab.

Buckley: Wow.

Franceschini: And while I was working, it was not the same. I couldn’t bring myself to commit. My life was about three months by three months. I was dedicated to finishing what I started, making sure my students, my postdocs, get advancing careers – more than thinking about: OK, I have to think about what comes next. Because I was afraid about thinking more than three months ahead. So, for the longest time, I didn’t submit any grants, just published a lot of papers, trying to finish everything. Helped junior faculty increase their visibility and such. And I’m glad I did that. I’m not glad that I didn’t write grants because after a while I realized I’m not going anywhere. I’m here. If I don’t have a grant I don’t have a salary. Because while I love working at MGH, MGH is not like, OK, I can do more teaching. There is no teaching for me. I’m not a doctor. My salary is 100% based on grants. And there is no sabbatical or anything. There is no safety net for me and for my lab. So, I had to re-start writing grants. And hopefully, some will come up.

Buckley: Yeah. I think about that all the time how as researchers in medical schools, there’s no safety net. You know if something happens and you can’t write grants, it’s just – it’s scary.

Franceschini: I didn’t realize it before. And definitely, as soon as I stabilize the lab, I will definitely spend time advocating for that. Because I found it terrible. I remember when I was younger and had a son, MGH has the Claflin Award to help women – young women. There are so many things helping you at every stage. But then if you are a professor… There is not a single internal grant I can apply to. There is no, is only – thank god - I have James Brink and Bruce Rosen on my side and they support me. But is at the department level it is not at the institution level. And it would be nice if there is something more formal. So, I’m not here to preach. I know young people need more. But I mean, things like this can happen, and thank god I’m functioning, but I may be not functioning enough, but I’m not ready to retire.

Buckley: Right, of course. And this could happen – I mean, you’ve got many more years of research productivity. I mean, this should be something that – yeah, I agree, there should be some mechanism –

Franceschini: No, there is nothing, at the NIH – nothing.

Buckley: Yeah. I found even when I took FMLA, I supported my own salary with my grant money to take time off. Right? There’s just something wrong with that.

Franceschini: Yeah, it’s like we work work work, but if we don’t get grants, we don’t get paid.

Buckley: That’s right. Yep. So, you’ve spent years studying patients who are critically ill in various capacities, and now you were the patient. What was it like being on the other end? And how did that experience change how you approach your studies?

Franceschini: Definitely it was not easy for my doctors. It’s never easy for my doctor. Because like the day the study on TAGRISSO was released in Paris at a conference, I had the picture of the screen of the results with the lines going down. So definitely, every time I met with the oncologist, there’s a lot of questions. And many times I’m like, “oh, I’m not sure. I don’t know.” And they’re great.

So, there is one thing that scares me: the medicine I’m taking is EGFR [epidermal growth factor receptor]. It blocks endothelial cell growth, so it has a lot of side effects, of course. There is a side effect that – I tried to call the company even, but nobody took me seriously – I see that is, experimentally with NIRS: it changes the elasticity of my cerebral blood vessel. So, I know I’m losing elasticity and I’m losing compliance. And I know this – if I will ever age, and I don’t think I will – it will bring to cognitive decline. It’s kind of scary. So right now, I’m taking it for three years. But I know if I take it only for three years, in two more years, I may have cancer again. There is the possibility I’ll continue to take it, but then what will happen to my cerebral blood vessels? Nothing comes for free or without consequences.

And definitely, something that – so I started diverting my studies more in about the interaction between cognitive decline and vascular function and cerebrovascular reactivity and such. I started two projects collaborating with people, and I would love, sooner or later, to also study the effect of this and other medicine on that. The problem – people usually don’t age if you take these medicines, so I don’t know even if it’s that useful. But like there are other side effects and I don’t see why this one shouldn’t be listed.

Buckley: Yeah, I mean, I do a lot of work with sickle cell disease. And when you have these kind of rare diseases, I think the patient advocates are the ones who make the most progress. So I mean, it’s so – it’s typical Mari, that you measured yourself!

Franceschini: I shouldn’t. I know I shouldn’t.

Buckley: No, but I mean, you just are constantly curious and you found that out.

Franceschini: Yeah, you end up not sleeping.

Buckley: It could lead to a whole new research direction. So, I was going to ask how it changed – or if it shaped your research directions at all? And it sounds like it has. And what about your outlook on existing projects? Has that changed at all? Or the directions that you’re taking?

Franceschini: I have less patience. So, if something doesn’t work that much, I don’t spend too much time on it. It’s like I’m running out of time. I’m more about application now, the hardware – I’m kind of tired of hardware development. I would like to move more on the patient side. But for the rest, no. I’m lucky that I have great support here. Stefan Carp has been supporting all the stuff that I don’t have the patience to continue. So, thank god I have him! He’s been awesome.

Buckley: That’s interesting that you say that you’re moving away from hardware. Well, it’s interesting for two reasons. One, because you guys are cranking out publications about new hardware approaches. But two, my next question was going to be, you’ve had this almost 30 years in the diffuse optics field, and so I’m wondering if you could share what you think the future of diffuse optics looks like to you? And what are the most promising avenues that you think people should be investing their time and energy into them?

Franceschini: The picture is great. I mean, the field is growing a lot. The technology is advancing very quickly and allows you to do things that were not possible a few years back. Definitely, fNIRS – with all these wearables and studies in neuroscience in a realistic place while doing things – is a new unique tool that you can only do with NIRS.

And on the clinical side, with all the telehealth, all the community health, all the low-resource settings, rural hospitals, and such, NIRS can help a lot. More wearable, more low cost, but still a strong diagnosis. So definitely, I don’t do a lot of fNIRS functional studies, but definitely, I’m very interested and drawn by the wearable telehealth and such.

Buckley: So, what advice do you have for a young trainee who is entering into the diffuse optics field today?

Franceschini: Oh my. In general, it’s not just about fNIRS or diffuse optics. They have to be passionate and be sure that that’s the avenue they want to take. And if they – so try to find a working place where they do the stuff they’re interested in. Other advice: if they are into fNIRS, they should get active in the fNIRS community. Many years ago, the way people learned about you and met you – it’s a conference. Now, it’s much more difficult, but you can get your name through the fNIRS community by participating in various committees. Organizing little things and using social media for work.

Buckley: And to get involved, that would entail the joining the Society for Functional Neuro –

Franceschini: fNIRS. Yeah, so it will entail – for example, there is the Communication Committee. And so every few months, you can write about what happened in one place. Or there is the Educational Committee. You can help organize workshop, seminars. You can volunteer to give a seminar. Rickson (Mesquita) is doing monthly seminars. So, expose yourself using these new tools. And there is the Diversity-Equity Committee. There is a lot of things. Help with the biennial meeting. There were a lot of young people that helped me organize the last meeting and it was so important to have their help. It’s a member-based community. People learn about you and they learn you are the next person in line to grow on the field. It’s not just because of your publications, but also for your dedication to grow the fNIRS community and to spread the voice about fNIRS and such.

Buckley: Some really great advice. I hadn’t thought about how much times have changed. I mean, the pandemic did yield some positive change in that regard, right? I mean, maybe that change would’ve come pandemic or not, but it certainly accelerated things.

Well, finally, in our last few minutes, certainly through your role in the presidency of SfNIRS, and just in generally, you’ve been a staunch advocate for female and underrepresented minority trainees and helping them advance their carriers. Do you have any pearls of wisdom for trainees who come from historically underrepresented groups?

Franceschini: Oh my, I’m not sure I have a word of wisdom. I just I want to make them understand that they are relevant. They are important. It’s not because they are from an underserved or underrepresented community, but because they see things in a different angle. If you want to advance in science, you have to pose different questions and look at things in different ways. They are really important for the growth of any field, so they shouldn’t compromise. They should find a school, a lab, where they are respected and valued for their diversity, for the fact that they think in a different way or see things in a different way and make progress toward things that – with a all-male scientists, white scientists – ther will not be done. So, there is really need for diversity in science in any field. And there are several opportunities out there right now, finally. So take any opportunity that comes! Don’t let them pass. And stay informed and advocate for yourself.

Buckley: That’s great advice. Well, thank you so much, Mari, for your time.

Franceschini: Thank you, Erin.

Buckley: Thank you – it’s been so fun! I’ve learned so much and it’s been great to sit and talk with you.
